# Evaluating Mindful With Your Baby/Toddler: Observational Changes in Maternal Sensitivity, Acceptance, Mind-Mindedness, and Dyadic Synchrony

**DOI:** 10.3389/fpsyg.2019.00753

**Published:** 2019-04-24

**Authors:** Moniek A.J. Zeegers, Eva S. Potharst, Irena K. Veringa-Skiba, Evin Aktar, Melissa Goris, Susan M. Bögels, Cristina Colonnesi

**Affiliations:** ^1^Research Institute of Child Development and Education, University of Amsterdam, Amsterdam, Netherlands; ^2^UvA Minds, Academic Outpatient (Child and Adolescent) Treatment Center, University of Amsterdam, Amsterdam, Netherlands; ^3^Department of Clinical Psychology, Leiden University, Leiden, Netherlands; ^4^Department of Developmental Psychology, University of Amsterdam, Amsterdam, Netherlands

**Keywords:** mindful parenting, mother–child interaction, maternal sensitivity, mind-mindedness, emotional communication, early intervention

## Abstract

Studies on the effectiveness of mindful parenting interventions predominantly focused on self-report measures of parenting, whereas observational assessments of change are lacking. The present study examined whether the Mindful with your baby/toddler training leads to observed changes in maternal behavior and mother–child interaction quality. Mindful with your baby/toddler is a 8- or 9-week mindful parenting training for clinically referred mothers of young children (aged 0–48 months), who experience parental stress, mother–child interaction problems, and/or whose children experience regulation problems. The study involved a quasi-experimental non-random design including a sample of 50 mothers who were diagnosed with a mood disorder (*n* = 21, 42%), an anxiety disorder (*n* = 7, 14%), post-traumatic stress disorder (*n* = 6, 12%), or other disorder (*n* = 7, 14%). Mothers completed a parental stress questionnaire and participated in home observations with their babies (*n* = 36) or toddlers (*n* = 14) during a waitlist, pretest, and posttest assessment. Maternal sensitivity, acceptance, and mind-mindedness were coded from free-play interactions and dyadic synchrony was coded from face-to-face interactions. Sensitivity and acceptance were coded with the Ainsworth’s maternal sensitivity scales. Mind-mindedness was assessed by calculating frequency and proportions of appropriate and nonattuned mind-related comments. Dyadic synchrony was operationalized by co-occurrences of gazes and positive facial expressions and maternal and child responsiveness in vocal interaction within the dyad. Coders were blind to the measurement moment. From waitlist to pretest, no significant improvements were observed. At posttest, mothers reported less parenting stress, and were observed to show more accepting behavior and make less nonattuned comments than at pretest, and children showed higher levels of responsiveness. The outcomes suggest that the Mindful with your baby/toddler training affects not only maternal stress, but also maternal behavior, particularly (over)reactive parenting behaviors, which resulted in more acceptance, better attunement to child’s mental world, and more “space” for children to respond to their mothers during interactions. Mindful with your baby/toddler may be a suitable intervention for mothers of young children with (a combination of) maternal psychopathology, parental stress, and problems in the parent–child interaction and child regulation problems.

## Introduction

In Western society and in today’s media, the transition into motherhood (or having another baby) is represented as a joyful and exciting time as this is assumed to be a period of emotional growth that emerges naturally (Winson, 2009). For many mothers, this idealistic image is not a close representation of their experience of this transition, as having a baby can be stressful and challenging (Ben-Ari et al., 2009; Kwon et al., 2013). Stress in mothers involves the extent to which mothers perceive themselves as having access to the resources required to carry out the parenting role (Belsky, 1984). Mothers of newborn children often juggle between holding on to their old life and adapting to newly gained responsibilities, including the regulation of the child sleeping and eating pattern, continuous availability, and regular worries about their infants’ health and development (Hung, 2007). These newly gained responsibilities affect career paths, sleeping patterns, romantic relations, and identities, that can get lost in the role of being a mother (Dew and Wilcox, 2011; Epifanio et al., 2015). Further, toddlerhood places distinctive tasks and challenges on parents with regard to the different developmental needs of children, such as the onset of independence, willfulness, and social competence (Edwards and Liu, 2002; Kwon et al., 2013). Thus, whereas being a mother is expected to bring joy, motherhood in the early years also brings distress upon a lot of mothers.

Elevated or recurrent levels of stress can lead to chronic stress, which increases the risk of mental health problems (Lupien et al., 2009). A remarkable high percentage of the new mothers develops depression (19.2%) or anxiety disorder (11.1%) in the first 3 months after child birth (Gaynes et al., 2005; Reck et al., 2008), and during toddlerhood elevated stress levels continue to predict depression and anxiety (Mathiesen et al., 1999). Stress and mental health problems are not only harmful to caregivers themselves, but also to children. The high rate of psychopathology and impaired functioning in the offspring of caregivers with, for instance, anxiety or depression, compared with caregivers without mental health problems is one of the best reproduced findings in psychiatry (e.g., Eley et al., 2015; Weissman et al., 2016). Anxious, depressed, or highly distressed parents have shown to lack frequent mentalizing and sensitive parenting behaviors during interactions (Nicol-Harper et al., 2007; Feldman et al., 2009; Pawlby et al., 2010; McMahon and Meins, 2012), which may evoke poor quality of parent–child interactions (Crnic et al., 2005). Low-quality interactions, in turn, impede the child’s optimal development and increases the risk of socio-emotional problems, such as perceived temperamental difficulties and insecure attachment representations (Crnic et al., 2005; Henrichs et al., 2009). Understanding how we may prevent or reduce parental stress seems therefore an important goal for mental health care sciences.

Mindfulness is awareness that arises through paying attention in the present moment to whatever appears and observing it non-judgmentally and without reactivity (Brown and Ryan, 2003; Kabat-Zinn, 2003; Creswell and Lindsay, 2014). Practice in mindfulness meditation have been shown to be effective in improving stress regulation (Khoury et al., 2015). The past two decades, the application of mindfulness in the context of parenting stress (i.e., mindful parenting) is growing (Bögels et al., 2010). Mindful parenting interventions are relationally oriented and aim to stimulate parents to focus mindful attention on parent–child interactions (Cohen and Semple, 2010). During mindful parenting training, parents learn to observe and listen to their child in a special way: deliberately, with full attention, and without judgment. Further, they learn to recognize and to make a distinction between their own emotions and those of the child, to lower parental reactivity in parent–child interactions, and to feel compassionate for themselves and their child (Duncan et al., 2015).

An adaptation of mindful parenting addressing mothers who experience stress in taking care of their young children is the Mindful with your baby/toddler training (Potharst et al., 2017, 2018). Mindful with your baby/toddler is a group training (Bögels and Restifo, 2013), involving meditation exercises based on mindfulness-based stress reduction training (MBSR; Kabat-Zinn, 1990), and mindfulness-based cognitive therapy (MBCT; Segal et al., 2002, 2012). The training is adapted to the context of parenting in early childhood and to the presence of the young children in the training. Other important elements of the training are inquiry, in which participants share their experiences during mediations, and psycho-education about themes related to both mindfulness and child development (i.e., the circle of security is introduced as a frame of reference for looking at attachment-related behavior of the children; Powell et al., 2013). In the Mindful with your baby/toddler training, parents not only learn to increase their awareness of inner experiences in the present moment, but also in the presence of, and in relation to their child. They learn to be attentive to their child and the child’s signals, and practice mindfulness in stressful situations (Potharst et al., 2017, 2018). Having their child by their side during the training (in most of the sessions) helps mothers to apply what they learn during training to daily life experiences with their child.

Two previous studies on the effects of the Mindful with your baby/toddler training on mother and child outcomes showed positive effects on a wide variety of mother and child outcomes (Potharst et al., 2017, 2018). In the first study including 37 mothers and their 0 to 18-months-old infants, mothers reported significantly higher scores on questionnaires on mindfulness, self-compassion, mindful parenting, as well as on well-being, psychopathology, parental confidence, responsivity, and hostility at posttest, 8-week follow-up, and 1-year follow-up (Potharst et al., 2017). In the second study (Potharst et al., 2018), including 18 mother–toddler dyads (aged 18–48 months), mothers reported positive changes in child psychopathology, mindfulness (awareness and non-reactivity), and self-compassion and these changes sustained or further improved during the follow-up period. Further, mothers reported lower levels of child dysregulation, maternal internalizing psychopathology, maternal stress, sense of incompetence, and higher levels of non-judging of inner experience, but only at the 2- and 8-months follow-up. Mothers also showed more sensitive and accepting behaviors during observations at posttest in this study.

These two studies provided first indications that the Mindful with your baby/toddler training may be beneficial, not only for the mother, but also for the mother–child relationship. However, the results on the mother–child relationship were either based on a small sample size (*n* = 18) of mother–toddler dyads, or based on maternal self-report, while this is not sufficient to measure parent–child interaction (Miron et al., 2009). When investigating change in complex transactional relationships such as the mother–child relationship, survey data may be biased by social-desirability of participants, or bias in interpretations of questions, and limitations with regard to the operationalization of complex relational constructs (Hops et al., 1995; Dishion and Granic, 2004; Morsbach and Prinz, 2006). Since mindful parenting interventions are designed to bring about changes in the parent–child relationship, observational measures of both parenting behavior and the parent–child relationship quality should be included in effectiveness studies (Duncan et al., 2015).

In the present study we, therefore, investigated the effects of the Mindful with your baby/toddler training observing different features of parenting behaviors and the interaction quality between mothers and their child. More specifically, we have focused on the following dimensions that have been shown to be particularly important for children’s early development and that are likely to change from mindful parenting training: parental sensitivity, acceptance, mind-mindedness, and dyadic synchrony. Below, we first briefly explain these parenting behaviors and characteristics, as well as their importance in predicting adaptive child development. We then explain why and how mindful parenting training in general, and the Mindful with your baby/toddler training in particular, might lead to changes in these behaviors and characteristics.

*Parental sensitivity* refers to the parent’s ability to interpret the child’s (behavioral, physical, and emotional) signals and respond to them in an appropriate and prompt manner. This concept has grown out of observational research attempting to understand variations in children’s secure attachment to their parents (Ainsworth, 1969; Ainsworth et al., 1974, 1978). Sensitivity is assessed from home-based observations of parent–child interaction, by rating the entirety of parenting behaviors shown during the interactions on a scale from 1 to 9 (Ainsworth et al., 1974). From the same home observations, Ainsworth (1969) developed a scale of *acceptance versus rejection*. A parent is accepting when there is sufficient balance between positive and negative feelings of the parent toward the child. The accepting parent respects the child’s desire for autonomy, mastery, and negative emotion (anger and frustration). Acceptance furthermore encapsulates the parent’s ability to empathize with the child, without losing touch with his or her own positive and negative emotions (Ainsworth, 1969). The importance of sensitive and accepting caregiving with regard to children’s adaptive and healthy development has become clear from a large body of research over the past decades. Parental sensitivity and acceptance have shown to predict a wide variety of positive child outcomes, most important children’s secure attachment, affect/stress regulation, and social–emotional competence understanding (e.g., Volling et al., 2002; Hughes et al., 2005; Khaleque and Rohner, 2012; Putnick et al., 2015; Taylor-Colls and Pasco Fearon, 2015; Zeegers et al., 2017).

*Mind-mindedness* is defined as parents’ tendency to treat their child as a mental agent, an individual with autonomous thoughts, feelings, and desires (Meins, 1997, 2013). This concept also grew out of observational research attempting to understand variations in (in)secure child–parent attachments (Meins, 1997; Meins et al., 2001). In early childhood, mind-mindedness is assessed as parents’ tendency to comment appropriately or in a nonattuned manner on their infant’s presumed internal states during a free-play situation (Meins et al., 2001; Meins and Fernyhough, 2015). The appropriate and nonattuned indices reflect two orthogonal dimensions of mind-mindedness, unrelated to each other in mothers (Meins et al., 2003, 2012). Appropriate mind-related comments reflect attunement to and validation of the infant’s internal state. Nonattuned comments reflect the extent to which misinterpretations of the infant’s state emerge, and/or when parents project their own state of mind or impose their own agenda on the infant (Meins, 2013). Greater mind-mindedness is indicated by high levels of appropriate mind-related comments or low levels of nonattuned mind-related comments. Mind-mindedness has shown to be lower in mothers with mental disorders, mothers who experience parenting stress, and in adolescent mothers (Pawlby et al., 2010; McMahon and Meins, 2012; Crugnola et al., 2014). Moreover, next to sensitivity, mind-mindedness has also shown to be an important and independent predictor of secure attachment, emotion regulation, social-emotional functioning in early childhood (Meins et al., 2002; Laranjo et al., 2008; Bernier et al., 2010; Zeegers et al., 2017, 2018).

*Dyadic synchrony* involves the co-occurrence and coordination of attention (gaze), emotional expressions, and vocalizations during the parent–child interaction (Yale et al., 2003; Colonnesi et al., 2012; Beebe et al., 2016). The general concept of dyadic synchrony refers to an array of interactive behaviors between parent and child such as responsiveness, reciprocity, mutuality, and shared emotion, typically assessed during face-to-face interactions. In the present study we focus on two forms of parents’ and children’s temporal coordination of behaviors. First, the temporal contingency of facial expressions and gaze (Yale et al., 2003; Colonnesi et al., 2012). Second, the turn-taking in vocal interaction (Feldstein et al., 1993; Gratier et al., 2015; Beebe et al., 2016), assessing how often the vocalizations of the mother were followed directly by vocalizations of the child and vice versa. Both the synchronous timing and the vocal turn-taking are considered to be important determinants of the quality of early parent–child interaction. That is, both provide children with opportunities to experience the mutual regulation of positive arousal, and to construct the structure of contingency and coordination characteristic of adult communication (Feldman et al., 1999; Leclère et al., 2014). Symptoms of depression, anxiety, and distress in mothers were shown to be related to disturbances in dyadic synchrony (Feldman, 2007), which is directly linked to infants’ current and later social, emotional, and psychological functioning (Feldman et al., 1999; Moore and Calkins, 2004; Feldman, 2007; Lindsey et al., 2009; Leclère et al., 2014).

Considering the core elements of mindful parenting interventions, and more specifically the core elements of the Mindful with your baby/toddler training, there are several reasons why it is important to study the effects of training on mothers’ sensitivity, acceptance, mind-mindedness, and dyadic synchrony. First of all, the Mindful with your baby/toddler training involves practice in listening to the child with full attention through mindfulness meditation (Potharst et al., 2017). These practices are thought to improve parents’ attention and receptive awareness to the experiences of the present moment (Brown and Ryan, 2003; Baer and Krietemeyer, 2006). The mindfulness meditations in Mindful with your baby/toddler also aim to improve parents’ self-control and to reduce their immediate reactions to their own thoughts, or feelings and external child-related events. Additionally, parents get the opportunity to practice being attentive to their own and to the child’s inner states by means of individual, and mother–child watching meditations, as well as the inquiry afterward (Siegel and Hartzell, 2003). These mindful parenting abilities all underlie parents’ tendency to form correct interpretations of children’s behavioral and verbal signals. That is, they reduce the use and influence of automatic cognitive processes, preventing bias in the interpretations of signals (Duncan et al., 2009). In turn, an appropriate interpretation of the child’s signals is at the heart of the concepts of maternal sensitivity and mind-mindedness (Ainsworth et al., 1974; Meins et al., 2001; Meins, 2013). Therefore, mothers are expected to show less insensitive behaviors and greater levels of mind-mindedness after the training.

Another important focus of the Mindful with your baby/toddler training is teaching parents to take a non-judgmental and compassionate stance toward their child’s and their own traits, attributes, and behaviors, which leads to the lower rejecting and dismissing parenting behaviors, as well as respect for the child’s autonomy (Ainsworth, 1969; Duncan et al., 2009; Bögels and Restifo, 2013). We, therefore, expect that after the training mothers will be more accepting as rated by independent observers. Furthermore, higher levels of compassion for the self and child should also come forward in positive changes in parental acceptance, as more self-compassion would lead to more positive, and less negative, affection in the parent–child relationship (Ainsworth, 1969).

Lastly, the above described mindful parenting behaviors and abilities can also lead to more implicit and embodied forms of attuned caregiving. As mindful parents are sensitive both to the content of conversations as well as their child’s tone of voice, facial expressions, and body language (Duncan et al., 2009), this might also be reflected in more synchronous timing of facial expressions and gazing (Siegel and Hartzell, 2003). We, furthermore, expected that mothers would show less turn-taking behaviors, as they were stimulated to be attentive to the present moment, in a non-judgmental and non-reactive manner. Additionally, we expected that children would show higher levels of turn-taking (responsiveness) as a result of increases in mothers’ mindful attitude and lower (over)active parenting during mother–child interactions.

The present study evaluated the effects of the Mindful with your baby/toddler training for mothers of young children (aged 0–48 months), who experience parental stress, mother–child interaction problems, and/or whose children experience regulation problems. A quasi-experimental design was used, with a waitlist assessment, pretest, and posttest. On the basis of the above-mentioned literature, we hypothesized that the Mindful with your baby/toddler training would be effective in reducing parenting stress, but also in improving observed maternal sensitivity, acceptance, mind-mindedness, and mother–child synchrony.

## Materials and Methods

### Study Design and Procedure

The present study had a quasi-experimental design, consisting of three measurement waves (waitlist, pretest, and posttest). During these waves home visits were conducted to record mother–child free-play sessions and face-to-face interactions. Furthermore, mothers filled out online questionnaires on their levels of parenting stress. The waitlist assessment was administered at least 5 weeks before starting the Mindful with your baby/toddler training. The mean waiting time for those who had to wait was 7.60 weeks (*SD* = 1.30). The home observations were repeated the week before the start of the training (pretest), and the week directly after the training (posttest). The home observations were coded by trained coders who were blinded to the measurement occasions (waitlist, pretest, and posttest).

Data of the present study were collected from 15 group trainings, which consisted of three to six mother–child dyads and started between October 2015 and February 2018. The intervention took place at a community child mental health center or a mindfulness center. Fifty mothers with their infants (*n* = 36) or toddlers (*n* = 14) were admitted to Mindful with your baby/toddler because of parental stress and/or mother–child interaction problems and/or child regulation problems. They were referred by general practitioners, midwives, or mental health care providers or they could enroll themselves.

Mothers were asked to participate in this research before the start of the training and gave informed consent. The study was approved by the ethical committee of the Faculty of Social and Behavioral Sciences at the University of Amsterdam. The mother–toddler dyads that took part in the current study were also part of an earlier study on the self-reported effects of the Mindful with your toddler training (Potharst et al., 2018). Part of the data on sensitivity, acceptance, and parenting stress was also presented in this article.

### Instruments

#### Parenting Stress

Parenting stress was assessed with the Dutch Parenting Stress Index-Short Form (PSI-SF, Brock et al., 1992), based on the American Parenting Stress Index (Abidin, 1983). The Dutch PSI-SF originally consists of 25 item, for example, “Considering only this child, parenthood is more difficult than I thought it would be.” Items are rated on a 6-point Likert scale, ranging from 1 (totally disagree) to 6 (totally agree). We removed two items, since they were not suitable for measuring parenting stress within the infant–caregiver relationship (i.e., “My child’s attention fades more often than I thought” and “When I prohibit something, later, my child will do this again”). In the analyses, we used mothers’ average item score as outcome measure (i.e., sumscore divided by 23). The Dutch PSI possesses good reliability, with reliability estimates ranging between α = 0.92 and α = 0.95 (Brock et al., 1992; Egberink et al., 2014). In the present study, internal consistency for the total score at pretest was α = 0.92.

#### Sensitivity and Acceptance

Sensitivity and acceptance were assessed from the 10-min free play sessions recorded at home. Mothers were instructed to play with their child with (5 min), and without (5 min) age-appropriate toys. Both scales were assessed using the scale of Ainsworth (1969). The first scale, *sensitivity versus insensitivity*, captures whether a mother is sensitive or insensitive to the signals of her child. Sensitive mothers made themselves available to perceive child signals, attributed meaning to these signals by acting promptly and appropriately upon them. For instance, a low score was given when a mother initiated a new toy when the child was still actively engaged with another toy. The second scale, *acceptance versus rejection*, captured whether a mother showed acceptance of the child’s initiatives and positive and negative feelings, while showing patience, positive affectivity, and warmth toward the child. For instance, a low score was given when mothers told their children to be quiet when they started crying. Video-observations were coded by four trained coders who evaluated every free-play session by assigning a score from 1 (highly insensitive/rejecting) to 9 (highly sensitive/accepting). Twenty percent of the observations were coded to assess inter-rater agreement. The intra-class correlation (ICC) among the coders was excellent (ICC = 0.83) for the *sensitivity versus insensitivity* scale and good (ICC = 0.76) for the *acceptance versus rejection* scale (Cicchetti, 1994). To prevent bias from single raters, every video-fragment was coded twice, by two different observers. Differences in scores were resolved by discussion.

#### Mind-Mindedness

Mothers’ mind-mindedness was assessed from the same 10-min free-play session as used to assess maternal sensitivity. Each spoken word or sentence of the mother was transcribed and coded by two independent observers using a translated version of the mind-mindedness coding manual (Meins and Fernyhough, 2015). The mind-related comments were categorized according to the specific state the parent referred to. Categories were *cognitions* (e.g., “you recognize this toy from home”), *likes and dislikes* (e.g., “you don’t like this ball”), *emotions* (e.g., “you’re excited to play with these toys”), and *epistemic states* (i.e., “are you teasing me?”). Comments that were obviously meant to be dialogue said/thought by the infant (e.g., “Mommy, can you help me?”) were also classified as mind-related.

Second, mind-related comments were classified as being appropriate or nonattuned. Appropriate comments are those for which: (a) the trained coder agreed with the parent’s reading of the infant’s internal state, (b) the internal state comment linked the infant’s current activity with similar events in the past or future, or (c) the parent voiced (using the first person) what the child might say if he or she could speak. Comments were classified as nonattuned when the coder believed (a) the parent misread the internal state of the child, or (b) the comment referred to a past or future event that had no obvious relation to the infant’s current activity (e.g., “I’m sure you would like to feed the ducks later”). We calculated mind-mindedness in terms of the frequencies of mothers’ appropriate and nonattuned mind-related comments. Additionally, in order to control for maternal verbosity, we calculated proportions of mind-related comments by dividing the total amount of appropriate or nonattuned comments by the total amount of comments a mother made during the free-play session (Meins and Fernyhough, 2015).

Twenty percent of the observations was randomly selected to calculate the inter-rater agreement. The inter-rater agreement was κ = 0.97 for mind-related comments and κ = 0.87 for appropriateness of mind-related comments, which can both be classified as “almost perfect agreement” (Landis and Koch, 1977). Disagreements were resolved by discussion.

#### Dyadic Synchrony

In order to observe dyadic synchrony, 4-min face-to-face interactions were recorded (Tronick et al., 1978). The child was placed in a seat in front of the mother (keeping a 30–50-cm distance), and the mother was instructed to talk to and play with her child, as she would normally do at home, without objects. A dual lens camera recorded both the mother’s and the infant’s face and upper body. Three trained observers coded infants’ gaze direction facial expression and vocalizations independently of one another on a 1 s time base (state event; event with a start time and an end time) using *The Observer XT 13.0* (Zimmerman et al., 2009). The inter-rater agreement in this observation could also be classified as “almost perfect” (Landis and Koch, 1977): κ = 0.88 for gazing, κ = 0.89 for facial expressions, and κ = 0.87 for vocalizations. Dyadic synchrony was studied by examining the temporal coordination and the interactive contingency of the following three behaviors (Harrist and Waugh, 2002):

##### Gaze

The coding for children’s gaze included: (a) gaze at the parent when children were looking at their parent’s face or hands, and (b) gaze elsewhere referred to children looking away or non-observable looking. Similarly, the coding for mother’s gazing included: (a) gaze at the child when mothers were looking at their children’s face or hands, and (b) gaze otherwise referred to mothers looking away or non-observable looking. Gaze otherwise was not included in the further analysis, but it represents the remaining time of the observation (240 s).

##### Positive facial expressions

We coded the emotional valance of mothers’ and children’s facial expressions (positive, neutral, and negative). Earlier studies showed that in typical interactions mothers’ facial expressions are predominantly positive, and rarely and negative in face-to-face interactions (Aktar et al., 2017). If present, negative facial expressions often occur reflect the child’s negative affect. We, therefore, only examined the co-occurrence of positive facial expressions in the current study. In line with this earlier evidence, less than 1% of maternal facial expressions during pretest were negative in the current study. We coded positive facial expressions in terms of closed and open smiles identified by raising corners of the lips, constriction of the eyes, raising of the cheeks, and opening of the mouth (Ekman and Friesen, 1978; Messinger et al., 2001).

##### Vocalizations

Vocalizations included verbalizations (words or sentences) and vocalizations: positive vocalizations such as chuckling, giggling, or laughing; neutral vocalizations such as babble; and negative vocalizations such as crying or fussing. For the analyses, positive and negative vocalizations were added up to a total vocalization score. Vegetative and reflexive vocalizations (hiccups, coughs, burps, etc.) were not coded.

The singular behavior of mother and child and their time-based co-occurrences were computed using the software for the collection and analysis of observational data, The Observer. With regard to dyadic synchrony, the following co-occurrences of pairs of behaviors were coded: (a) *coordination of gaze:* temporal co-occurrence of child gazing toward mother and mother gazing toward the child (in seconds; Lotzin et al., 2015); (b) *coordination of positive facial expression*: temporal co-occurrence of mother and child both displaying positive facial expressions (in seconds; Riehle et al., 2017); (c) *coordination of positive facial expression during gaze:* temporal co-occurrence of children’s positive facial expression when gazing toward mother and mother’s positive facial expression when gazing toward the child (in seconds; Weinberg and Tronick, 1994). With regard to the turn-taking vocal interaction between mother and child, the following turn-taking sequences were coded: (a) *maternal responsiveness*, mother responds to child’s vocalization when the mother’s vocalization happens within 2 s after the child’s vocalization (frequencies; Lammertink et al., 2016); (b) *child responsiveness*, child responds to mother’s vocalization when the child’s vocalization happens within 2 s after the mother’s vocalization.

For the variables coordination of gaze, positive facial expressions, and positive facial expressions percentages were calculated dividing the duration of the behavior (in seconds) by the total duration of the observation ^∗^ 100. Percentages of *maternal responsiveness* were calculated by dividing the number of maternal vocalizations after child vocalizations by the total number of child vocalizations. Percentages of *child responsiveness* were calculated by dividing the number of child vocalizations after maternal vocalizations by the total number of maternal vocalizations.

### Intervention

The Mindful with your baby training and the Mindful with your toddler training are similar to each other in terms of aims, as well as in the mindfulness exercises. The training consists of eight (babies) or nine (toddlers) weekly sessions of 2 h, and an additional follow-up session 2 months later. The sessions are carried out in small groups with a maximum of six dyads per group. Each group is led by an experienced Mindful with your baby/toddler trainer (EP or IV). Other than the number of sessions, the infant and toddler training programs differ with regard to the presence of the children. In the Mindful with your baby training, the babies are present in all sessions, except for the first and the fifth session. The first session allows for a clear introduction in, and deeper understanding of mindfulness and the fifth session allows for a possibility to focus on learning self-compassion with full attention. In the Mindful with your toddler training, the toddlers join the training after Session 4, so from Session 5 to 9. The sessions without the toddlers are needed to lay a foundation in mindfulness abilities, before mothers are asked to apply these abilities with their toddler, which appeared to be more challenging in toddlers than in babies. Toddlers can make an appeal to their mothers quite strongly and directly, and this may make it harder for mothers to keep an observational stance while interacting with them. Also, toddlers explore more actively than babies, which brings about themes like conflicts between children, limit setting, shame about a child’s behavior, etc. The sessions with the children allow for mothers to directly apply their learned mindfulness skills when they are in their parental role, making what is learned in the training more generalizable to the parent’s everyday life.

The content of the training programs is described more elaborate in Potharst et al. (2017, 2018). Structural components of the training are formal mindfulness meditations based on MBSR (Kabat-Zinn, 1990) and MBCT (Segal et al., 2012). Another import component of the trainings involves meditations in which mothers focus on their child. This is done by watching meditations, in which mothers are asked to watch every step and behavior of the child with curiosity, and to empathize with the intentions and the discoveries of the child.

In the present study, trainers were accompanied by an Infant Mental Health Specialist (IMH-specialist) or psychologist in training. The IMH-specialist is responsible for the well-being of the mother–child dyads: she can observe the mother–child interaction, offer (emotional) support, and be available for discussion and evaluation with the trainer after the training sessions. However, for both IMH-specialists and the psychologists in training, the main task involved watching, and being available for the children during the meditation sessions in which the mothers close their eyes, and making sure the children were both emotionally and physically safe (e.g., by giving explanation of what happens to the children or by warning the mindful parenting trainer or a parent when the meditation lasts too long for a particular child). We examined whether the difference in professional training of the second trainer affected the outcomes (see the section “Results”).

### Data Analyses

The repeated measurements before and after the training led to a hierarchical dataset. We, therefore, used multilevel regression models consisting of repeated measurements of time (level 1), nested in mother–child dyads (level 2) to analyze the data. Next to accounting for nested data, an advantage of multilevel regression analyses is that missing data can be handled, and imputation is not needed (Kreft and De Leeuw, 1998). Analyses were ran with 50 families that completed at least the waitlist/pretest and posttest measures. Further, analyses were run with and without standardized scores on the continuous outcome measures. This way we could report on the unstandardized regression estimates (*B*) as well as the standardized estimates (β, which could be interpreted as effect size). The random effects of intercept and time on the outcome measure were tested in each model (*p* < 0.050). Additionally, to study if the treatment outcomes from the main multilevel analyses differed across the infant or toddler training, we reran the reported models after including the type of group (baby or toddler), and (in a separate model) the presence of second trainer (IMH specialist or psychologist in training), as well the interaction effect between time and group/trainer as covariates. Second, we tested whether adding random slopes to the models improved the fit of the model to the observed data, which would indicate that mothers show variation in their change from pre- to posttest.

To correct for the multiple comparisons, a false discovery rate (FDR) of 0.05 was applied (Benjamini and Hochberg, 1995). The FDR determines the expected proportion of false discoveries among significant findings, yielding a *q*-value based on the *p*-values of the multiple comparisons. *P*-values below the set *q*-value are considered statistically significant.

## Results

### Participants

Fifty mothers (*M*_age_ = 35.06 years; *SD* = 4.19) with their infants (*n* = 36; *M*_age_ = 9.57 months; *SD* = 5.38; 20 boys) or toddlers (*n* = 14; *M*_age_ = 2.50 years; *SD* = 0.57; 10 boys) participated in the Mindful with your baby/toddler training. Thirty-three children (66%) were firstborn. The mothers’ ethnicities were Dutch (*n* = 36; 72%), European-other (*n* = 3; 6%), and non-European (*n* = 11; 22%), and 22 (44%) mothers obtained a University degree, 23 (46%) a college degree, 2 (4%) secondary vocational education degree, and 2 (4%) a high school diploma. During the training, 24 mothers (48%) were working, 13 (26%) were on sick leave or without a job, 10 (20%) were stay-at-home mothers, 1 (2%) was a student, and 1 (2%) was on parental leave.

Based on clinical assessment during the intake sessions, mothers were diagnosed with a depression (21 mothers, 42%), anxiety disorder (17 mothers, 34%), post-traumatic stress disorder (PTSD) (6 mothers, 12%), or another disorder, such as an obsessive compulsive disorder or attention deficit hyperactivity disorder (7 mothers, 14%). Some mothers had more than one diagnosis. Fifteen mothers (30%) had no diagnosis. In the waitlist period, prior to the Mindful with your baby/toddler training, 62% (31 mothers) received psychological treatment or parenting support (often IMH treatment).

### Response Rates

**Figure 1:**
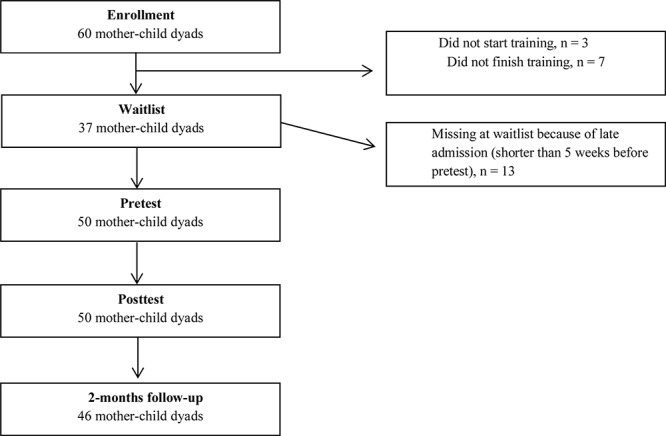
Flow diagram of the number of participants at each assessment time.

Figure 1 displays a flow chart of the participants at each measurement time. Three mothers did not want to participate in the home observations. For these mothers only demographic data and questionnaire data were available. With regard to the observational data, missing data on the mind-mindedness and sensitivity variables were due to technical problems or to mothers speaking a foreign language during the play. Missing variables on face-to-face interactions were more frequent due to technical difficulties or unclear recordings. In order to code synchrony in facial expressions and gaze, mother and child need to be recorded simultaneously by both lenses. Due to movement of the child and/or mother, some videos could not be coded due to poor recording. The dyads that did not have face-to-face recordings did not differ significantly from the dyads that did have recordings on any of the other observational measures at waitlist, pretest, and posttest. With regard to the data on sensitivity and mind-mindedness, 68% of the mothers were observed during the waitlist assessment, 92% during posttest, and 92% during follow-up. For dyadic synchrony, 50% of the mother–child dyads were observed during the waitlist assessment, 68% during pretest, and 68% during posttest. Exact numbers on the available data are presented in Table 1.

**Table 1:** Means and standard deviations of all observational outcome measures at three measurement occasions.

	Waitlist		Pretest		Posttest	
	*n*	*M* (*SD*)	*n*	*M* (*SD*)	*n*	*M* (*SD*)
**Mother report**						
Parenting stress	29	2.76 (0.83)	49	2.86 (1.00)	49	2.43 (0.83)
**Observations**						
Sensitivity	34	6.02 (1.75)	46	5.82 (1.88)	46	6.28 (1.85)
Acceptance	34	6.35 (1.91)	46	5.89 (1.93)	46	6.78 (1.59)
Appropriate mind-related comments (frequencies)	34	6.44 (5.72)	46	6.30 (4.49)	46	5.93 (3.73)
Nonattuned mind-related comments (frequencies)	34	2.71 (3.16)	46	2.41 (2.36)	46	0.91 (1.33)
Appropriate mind-related comments (%)	34	4.88 (3.66)	46	4.92 (2.75)	46	4.70 (2.78)
Nonattuned mind-related comments (%)	34	2.28 (2.49)	46	2.06 (1.98)	46	0.70 (0.10)
Coordination of positive facial expressions (%)	25	12.20 (12.15)	34	17.26 (15.38)	34	16.27 (17.03)
Coordination of gaze (%)	25	36.87 (22.87)	34	42.19 (23.32)	34	39.35 (24.69)
Coordination of positive facial expressions and gaze (%)	25	6.60 (8.60)	34	10.98 (9.80)	34	9.17 (12.11)
Child responsiveness (%)	25	15.48 (8.64)	34	16.00 (10.88)	34	20.14 (11.62)
Maternal responsiveness (%)	25	45.12 (20.23)	34	53.58 (24.70)	34	45.92 (18.34)

*Data are presented as mean (standard deviation), *n* = number of available cases*.

### Preliminary Analyses

The means and standard deviations of the outcome variables are presented in Table 1. The residuals of the analyses were normally distributed (Tabachnick and Fidell, 2013). We checked whether any of the outcome measures correlated with demographic variables of the mothers [age, educational level, nationality (Dutch/non-Dutch)] at pretest. Mothers with a higher educational level were rated as more sensitive and accepting than mothers with a lower educational level at pretest, *r* = 0.57 and *r* = 0.50. We therefore added educational level as a covariate to the analyses. We examined whether the analyses with and without educational level as a covariate yielded different results, which was not the case. Therefore, we report the results of the main analyses without educational level as a covariate.

### Effects of the Training

Table 2 presents the results of multilevel models with random intercepts of treatment outcome predicted by measurement occasion without any covariates. As we applied an FDR of 0.05, we reported the significance of effects in Table 2 when the *p*-values were below the set *q*-values. There were no significant changes on the outcome measures from waitlist to pretest. Mothers reported less parenting stress from pre- to posttest (small to moderate effect size). Compared to pretest, at posttest mothers were more accepting toward their child (small to medium effect size) and produced less nonattuned mind-related comments (large effect size). Children showed more responsiveness in turn-taking at posttest compared to pretest, as they were more likely to vocalize after the mother had vocalized (small to medium effect size). There were no pretest to posttest changes in the synchrony of facial expressions, gazing, and facial expressions during gazing.

**Table 2:** Unstandardized and standardized parameter estimates and *F*-values of multilevel models of observational outcomes predicted by measurement occasion (deviations from pretest).

	Waitlist			Posttest		
	*B* (*SE*)	β	*F*	*B* (*SE*)	β	*F*
**Mother report**			
Parenting stress	0.05 (0.13)	0.06	0.14	-0.24 (0.10)	-0.27	5.72*
**Observations**			
Sensitivity	-0.08 (0.28)	-0.04	0.74	0.43 (0.25)	0.24	2.93^†^
Acceptance	-0.36 (0.30)	-0.19	1.43	0.85 (0.27)	0.46	10.16**
Appropriate mind-related comments (frequencies)	0.21 (0.77)	0.05	0.08	-0.12 (0.69)	-0.03	0.04
Nonattuned mind-related comments (frequencies)	-0.26 (0.45)	-0.10	0.33	-1.50 (0.41)	-0.62	13.49***
Appropriate mind-related comments (%)	0.17 (0.55)	0.06	0.10	-0.06 (0.49)	-0.02	0.02
Nonattuned mind-related comments (%)	-0.23 (0.35)	0.12	0.45	-1.39 (0.31)	-0.70	19.63***
Coordination of positive facial expressions	4.81 (3.31)	0.32	2.12	-0.92 (2.97)	-0.06	0.10
Coordination of gaze	2.42 (5.46)	0.10	0.20	-1.57 (4.93)	-0.06	0.10
Coordination of positive expressions during gaze	3.09 (2.13)	0.30	2.11	-1.07 (1.91)	-0.10	0.32
Child responsiveness	0.87 (2.19)	0.09	0.16	4.24 (1.96)	0.40	4.67*
Maternal responsiveness	7.20 (4.79)	0.33	2.26	-7.61 (4.32)	-0.35	3.10^†^

*B, the unstandardized parameter coefficient of the waitlist, post-test, and follow-up relative to the pre-test; SE, standard error of parameter estimate; β, the standardized beta coefficient, ^†^*p* < 0.1, ^∗^*p* < 0.05, ^∗∗^*p* < 0.01, ^∗∗∗^*p* < 0.001. The parameter coefficients should be interpreted as relative to the pretest measurement*.

We added random slopes to each model to test whether mothers showed variation in their response to the intervention (i.e., some mothers might show more change than others). None of the random slope models showed an improved fit to the observed data.

### Covariates

We analyzed whether the treatment outcomes were dependent on the type of training group (baby or toddler) and/or whether the treatment outcomes were dependent on the presence of an IMH-specialist. There were no other significant interaction effects for type of group, suggesting that the outcomes described above apply to the mothers in the baby and toddler group. With regard to the presence of the IMH-specialist versus psychologist in training, we also did not find significant interactions effects.

## Discussion

Mindful with your baby/toddler is a group-based training for mothers of babies and toddlers who experience parental stress and/or problems in the parent–child relationship. The training is focused on reducing parental stress and improving the mother–child relationship through practicing mindfulness meditation with and without the child present. The main aim of this study was to evaluate whether the training not only reduces maternal self-reported parenting stress, but also changes objectively measured maternal behavior during parent–child interactions and mother–child interaction quality, as compared to waitlist. We therefore observed changes in maternal sensitivity, acceptance, mind-mindedness, and dyadic synchrony, next to collecting mothers’ parenting stress reports. The results showed that mothers reported less parenting stress after the training (small effect size), were more accepting (medium effect size), and made less nonattuned references to the child’s mental states (large effect size). The children showed higher levels of responsiveness after the training (small to medium effect size). No improvements occurred on any of the outcome measures after waitlist, suggesting that the training underlies the observed outcomes.

First as expected, maternal stress decreased after the training, indicating that the training is effective in reducing mothers’ stress in parenting their young children. The effect size however was small. In two earlier studies, parenting stress did not yet reduce at posttest but only 8 weeks after the Mindful with your baby/toddler training (Potharst et al., 2017, 2018), suggesting that parenting stress reductions may continue after the training has finished.

In line with our hypotheses, mothers behaved more accepting toward their children (small to medium effect size), which means that they showed less rejecting behavior in reaction to the child’s initiatives and positive and negative feelings, and a more positive, warm, patient, and non-reactive attitude. Maternal sensitivity did not improve significantly indicating that this mindful parenting training seems to tap into the core aspects of acceptance more than the core aspects of sensitivity. Indeed, when mothers practice mindfulness they increase their capacity of “being present” with whatever comes up, whether it is pleasant or unpleasant (Kabat-Zinn, 2003). Examples of something unpleasant during a formal meditation could be pain or worries, and mothers practice not only with becoming aware of these experiences, but also to meet them non-judgmentally and with equanimity. Further, in the mindful parenting exercises, mothers learn to generalize what is learned in interaction with their children. So, they learn to meet difficulties with their child, like crying, and their own inner reactions to such difficulties, with patience and kindness. In the training, mothers receive psycho-education about the fight, flight, and freeze stress reactions. They practice with becoming aware of their own stress-related action tendencies, applying mindfulness when they notice a stress reaction, and then making a conscious choice in how they want to respond to their child. Rejecting behavior is an example of a fight reaction that is directly addressed in the training, which aligns with the post-intervention changes in accepting behavior.

Mothers’ ability to postpone judgment and reaction may underlie the decrease in nonattuned mind-related comments. Especially when children show behavior that is challenging or confusing to mothers, they may tend to express their distress in the form of judgments about the child (e.g., saying “you always want to have it your way” or “you just want attention”). Or they may look for explanations of behavior aimed at finding peace in the difficult situation, rather than at staying open to what the child may be going through at that moment (e.g., “You are tired, it is time for your nap” when actually the child is frustrated because he is not allowed to touch something in the room). This tendency may be associated with parental experiential avoidance, which is an inability to tolerate their own internal distress in difficult parenting situations (Tiwari et al., 2008). Parental experiential avoidance may cause intrusive behavior in parents that is aimed at reducing the child’s distress or behavior, and thereby reducing the parent’s distress. In the Mindful with your baby/toddler training, mothers practice awareness in situations that are stressful for them and learn to notice not only their thoughts and feelings in such a situation, but also their tendency to act and deal with these feelings. They are also invited to become aware of “not knowing” why the child acts like he does or “not understanding,” and the distress that this may give, and to practice accepting this “not knowing.”

So possibly, the capacity to stay present in a non-judgmental way in the face of difficulty underlies both the improvement in acceptance and in nonattuned mind-related comments. On the other hand, the other dimension of mind-mindedness, appropriate mind-related comments, which did not improve in the current study, may be more related to encapsulate traditional notions of engagement, responsivity, and sensitivity (Meins, 2013; Zeegers et al., 2017). The question is whether there was no change in the extent to which mothers were inclined to interpret their child’s behaviors in terms of underlying mental states, or whether mothers did not verbalize these mind-related comments more often. In the watching meditation in which mothers practiced focusing their full attention to the child, they also practiced in reflecting on the experience of the child, but they were not invited to immediately verbalize these reflections. This is an important difference between mindful parenting training and a mentalization-based parenting program: the first focuses on awareness, while the latter focuses on the verbalizing emotions, intentions, and desires of the child (Sadler et al., 2006).

The mothers in the present study had proportions of nonattuned mind-related comments of 2–3% at waitlist and pretest, and 5% of the comments were classified as appropriately mind-related. In terms of frequencies, mothers made on average six appropriate mind-related comments and two to three nonattuned comments during a play session at the waitlist and pretest measurement. At posttest, mothers’ proportions of nonattuned comments decreased to 1% (frequency of 1 comment). Appropriate mind-related comments were still 5% (frequency of six comments). Unfortunately, there are no clinical or non-clinical norms of mind-mindedness available. We compared the mind-mindedness of the mothers in the present study with a non-clinical sample of Dutch mothers, who were living in the same urban area and had similar socioeconomic backgrounds (*n* = 116; Zeegers et al., 2018). In this study, proportions of nonattuned and appropriate mind-related comments at 12 months were 1% and 7%, respectively. These numbers indicate that at posttest, mothers’ mean levels of nonattuned mind-mindedness decreased to levels comparable in a non-clinical sample.

Turning to the results on dyadic synchrony, we found that children (both infants and toddlers) showed more vocalization after the mother vocalized, suggesting that they became more responsive to their mothers. These results may be explained better when considering the outcomes for mothers. That is, although non-significant, we found that mothers tended to show less responsiveness after the training (*p* = 0.087; small effect), possibly because they became less (over)reactive. We checked whether mothers talked less to their children from pre- to posttest. This was not the case. On average mothers made 127 comments both at pretest and posttest. Thus, it seemed that not mothers’ overall talk, but specifically their prompt reaction to the child’s vocalization decreased. These outcomes suggest that maternal reactivity decreased. Possibly, children showed more responsiveness at posttest because they experienced more “space” to react upon their mothers. There were no changes in the co-occurrences of positive facial expressions and gazing.

We studied the effects of the training for all training groups together, regardless of the age of the children. Our rationale was that both the baby and toddler training aim to reduce parenting stress and improve the quality of the mother–child relationship using the same methods: mindfulness meditation, watching meditation, psycho-education, and inquiry. We therefore hypothesized that in both baby and toddler groups maternal mind-mindedness, sensitivity, acceptance, and turn-taking behavior and dyadic synchrony would increase. Furthermore, by investigating the outcomes of the baby and toddler groups together, we increased statistical power. In order to study whether the training effects were different for the baby and toddler groups, we added interaction effects (Group × Posttest) to the multilevel models. These interaction analyses did not show that effects were different for mother–baby and mother–toddler dyads. However, future studies should replicate the present study, including a larger sample, in order to study possible differences in baby versus toddler groups in more detail.

A large proportion of the current study sample (almost 70%) was diagnosed with mood or anxiety disorders. These disorders are risk factors for mother–child interaction problems (Nicol-Harper et al., 2007; Bernard et al., 2018). However, treating maternal depression does not necessarily improve mother–child interaction (Forman et al., 2007; Kersten-Alvarez et al., 2011). A meta-analysis on the effectiveness of mindfulness-based interventions in participants with mood or anxiety disorders showed large effect sizes of mindfulness interventions on symptoms of anxiety and depression (Hofmann et al., 2010). Earlier studies on the effectiveness of the Mindful Parenting training in general (Bögels et al., 2014; Meppelink et al., 2016) and the Mindful with your baby/toddler training (Potharst et al., 2017; Potharst et al., 2018) showed that even if a mindfulness training is focused on parenting, it also decreases parental internalizing psychopathology. The behavior changes observed in this study imply that Mindful with your baby/toddler may be a suitable intervention for mothers who suffer from internalizing psychopathology and also experience problems in interaction with their baby or toddler, as both mother and child may profit from a Mindful with your baby/toddler training.

### Limitations and Future Directions

Some caution is warranted in interpreting the results. First of all, although the results of the waitlist period seem to suggest that the significant effects can be attributed to the training, conclusions about causality are limited by the lack of a randomized control group. Second, the effects of the training may be less generalizable to the entire population of Dutch mothers with stress. Mothers were referred to this training by general practitioners, midwives, a mental health care providers, or mothers signed up for the training themselves. All mothers were aware that they experienced parenting stress and were willing to learn mindfulness in order to learn to cope with their stress differently. It is unclear whether the selection of the present study’s participants affected the treatment outcomes.

Third, the age of the children that were included in this study varied, ranging from 4 months to 3.5 years. This relatively broad age range could have influenced the scoring of the different mother–child observations, particularly the scoring of maternal acceptance and sensitivity. That is, certain parenting behaviors were shown during mother–toddler observations only. For instance, boundary-setting behavior occurred during the mother–toddler interactions but hardly occurred during the mother–infant interactions. This means that sensitive and accepting behavior could have a different appearance depending on the age of the child. The training may have had an effect on parenting behaviors that were more likely to appear in the mother–toddler interactions than in the infant–mother interactions. We aimed to make the coding as unbiased as possible by double coding the recordings and blinding the observers to the measurement condition (waitlist/pretest/posttest). However, the age differences between the children could have biased the coding of sensitivity and acceptance.

Research studying observational effects of mindful parenting interventions is yet scarce. This study was the first to examine post-intervention changes in observed maternal sensitivity, mind-mindedness, and parent–child synchrony. With regard to future research, it might be interesting to compare the observed effects of the Mindful with your baby/toddler training with other interventions, such as a mentalization-based parenting program, and compare the outcomes of these interventions. We also recommend measuring the long-term effects of the Mindful with your baby/toddler training on observed changes in behavior, since mindfulness skills may require time for consolidation, independent practice, or generalization to the context of the parent–child interaction. Second, because of the limited sample size, we could not study the moderating or mediating effects of some variables. Analyses would have been seriously underpowered (Snijders and Bosker, 2012). This leaves a few questions unanswered. First of all, the present study did not take into account the influence of mother and child characteristics (e.g., temperament) that are known to – additively and interactively – contribute to parenting behavior (Achtergarde et al., 2015). Most important, while all mothers in this study suffered from elevated levels of stress, most mothers were also diagnosed with an anxiety disorder, depression, or PTSD. These (different) mental health problems could lead to differential effects of the training. Note, however, that Mindful with your baby/toddler has a transdiagnostic approach – the training is focused on changing repetitive, inflexible, distress-producing ways of thinking, perceiving, and behaving that are implicated in many disorders (e.g., anxiety, depression, posttraumatic stress, substance use, sleep disturbance, eating disorders, and chronic pain conditions; Greeson et al., 2014). We recommend that the present study is replicated in a larger sample of mother–child dyads in order to get a better understanding of whether and how mother and child characteristics influence the effects of the Mindful with your baby/toddler training.

Second, previous results suggest that a focus on the mental and emotional life of their child might give parents greater insight into the child’s behavior, thereby making it more comprehensible, meaningful, and predictable, and thus less likely to induce parenting stress (McMahon and Meins, 2012). This means that improvements in mindful parenting or mind-mindedness may moderate changes in maternal stress. To study these questions we recommend that the present study is replicated in a larger sample of mother–child dyads in order to get a better understanding of the working mechanisms of the Mindful with your baby/toddler training.

## Conclusion

The present study evaluated whether the Mindful with your baby/toddler training led to observed changes in maternal behavior and mother–child interactions. Mothers were found to be more accepting and show less nonattuned mind-related comments after the training, whereas children showed higher levels of responsiveness. These observational outcomes suggest that the Mindful with your baby/toddler training resulted in more accepting behavior, better attunement to child’s mental world, and more “space” for children to respond to their mothers during interactions. The Mindful with your baby/toddler training may be a suitable intervention for mothers who show a combination of parental stress, internalizing symptoms, problems in the parent–child interaction, and/or child regulation problems.

## Ethics Statement

The study was approved of by the Ethical Commission of the University of Amsterdam. All participants gave written informed consent.

## Author Contributions

MZ, EP, IV, EA, SB, and CC contributed to the design of the study. MZ did the statistical analyses. EP developed the trainings. EP and IV were the mindfulness trainers. MZ, IV, MG, and CC contributed to data collection. MZ, CC, and EP supervised data collection. MZ wrote the manuscript. All authors contributed to manuscript revision.

## Conflict of Interest Statement

SB is a shareholder of the clinic where the data collection took place, and published books about mindful parenting, and EP published a book in Dutch for parents about, and with the title Mindful with your baby. The remaining authors declare that the research was conducted in the absence of any commercial or financial relationships that could be construed as a potential conflict of interest.
